# Pathway Analysis of MicroRNA Expression Profile during Murine Osteoclastogenesis

**DOI:** 10.1371/journal.pone.0107262

**Published:** 2014-09-15

**Authors:** Tiziana Franceschetti, Neha S. Dole, Catherine B. Kessler, Sun-Kyeong Lee, Anne M. Delany

**Affiliations:** 1 Center for Molecular Medicine, University of Connecticut Health Center, Farmington, Connecticut, United States of America; 2 Center on Aging, University of Connecticut Health Center, Farmington, Connecticut, United States of America; University of Ulm, Germany

## Abstract

To design novel therapeutics against bone loss, understanding the molecular mechanisms regulating osteoclastogenesis is critical. Osteoclast formation and function are tightly regulated by transcriptional, post-transcriptional and post-translational mechanisms. This stringent regulation is crucial to prevent excessive or insufficient bone resorption and to maintain bone homeostasis. microRNAs (miRNAs) are key post-transcriptional regulators that repress expression of target mRNAs controlling osteoclast proliferation, differentiation, and apoptosis. Disruption of miRNA-mediated regulation alters osteoclast formation and bone resorption. Prior studies profiled miRNA expression in murine osteoclast precursors treated with RANKL for 24 hours. However, a more complete miRNA signature, encompassing early, mid and late stages of osteoclastogenesis, is wanting. An Agilent microarray platform was used to analyze expression of mature miRNAs in an enriched population of murine bone marrow osteoclast precursors (depleted of B220^+^ and CD3^+^ cells) undergoing 1, 3, or 5 days of RANKL-driven differentiation. Expression of 93 miRNAs, changed by >2 fold during early, mid, and late stages of osteoclastogenesis, were identified and sorted into 7 clusters. We validated the function and expression of miR-365, miR-451, and miR-99b, which were found in distinct clusters. Inhibition of miR-365 increased osteoclast number but decreased osteoclast size, while miR-99b inhibition decreased both osteoclast number and size. In contrast, overexpression of miR-451 had no effect. Computational analyses predicted mTOR, PI3 kinase/AKT, cell-matrix interactions, actin cytoskeleton organization, focal adhesion, and axon guidance pathways to be top targets of several miRNA clusters. This suggests that many miRNA clusters differentially expressed during osteoclastogenesis converge on some key functional pathways. Overall, our study is unique in that we identified miRNAs differentially expressed during early, mid, and late osteoclastogenesis in a population of primary mouse bone marrow cells enriched for osteoclast progenitors. This novel data set contributes to our understanding of the molecular mechanisms regulating the complex process of osteoclast differentiation.

## Introduction

The maintenance of bone homeostasis requires a tight control of the number and activity of osteoblasts, the bone-forming cells, and osteoclasts, the only cells able to resorb mineralized bone matrix. Osteoclast differentiation is an intricate process, regulated at multiple levels by transcription factors and post-translational modifications. In this process, myeloid progenitor cells differentiate into monocytes, commit to the osteoclast lineage, migrate, and then fuse into multinucleated polykaryons, at the expense of the alternative macrophage fate. Macrophage-colony stimulating factor (M-CSF, CSF1) and receptor activator of nuclear factor kappa-B ligand (RANKL) are key cytokines responsible for driving osteoclastogenesis from multipotential hematopoietic progenitors. Several intracellular signaling pathways promote commitment to the osteoclast lineage, and converge on the activation of NFATc1, the master transcriptional regulator of osteoclastogenesis. NFATc1, in combination with other transcription factors, including PU.1, MITF, NFκB, and c-Fos, coordinates the expression of genes necessary for bone resorption, such as Cathepsin K, Tartrate-resistant acid phosphatase (*Acp5, Trap*), and Calcitonin receptor [1]–[3].

More recently, a growing number of reports have demonstrated the important role of microRNAs (miRNAs) in osteoclast biology. miRNAs are short single-stranded, non-coding RNA that act, for the most part, as post-transcriptional regulators of gene expression. This is achieved primarily by binding target mRNAs at sites frequently located in the 3′ untranslated region (UTR). However, miRNA binding sequences have been identified also in the coding region and in the 5′ UTR [4], [5]. miRNA activity requires its incorporation in a RNA-induced silencing complex (RISC). Target recognition by the miRNA relies mainly on near-perfect complementarity of the mRNA with the miRNA “seed region”, a 6–8 nucleotide-long sequence in the 5′ end of the miRNA. Upon target binding, repression of gene expression is accomplished by suppressing translation, and/or decreasing the stability of the mRNA.

The critical role of the miRNA processing pathway in the osteoclast lineage was described. In vitro, silencing of DiGeorge syndrome critical region 8 gene (DGCR8), Argonaute2 (Ago2), and Dicer1, key miRNA processing factors, decreased osteoclastogenesis and bone resorption [6]. In vivo, deletion of Dicer in the myeloid lineage, using a CD11b promoter driven-Cre recombinase, and in mature osteoclasts, using a Cathepsin K promoter driven-Cre, led to the development of mild osteopetrosis, due to impaired osteoclast differentiation and activity [6], [7]. These studies highlight the overall importance of miRNAs in regulating osteoclast biology, and allude to their potential as therapeutic targets for pathologies caused by excessive or insufficient osteoclast activity. However, little is known about the function of individual miRNAs in osteoclastogenesis.

At present, few miRNAs and only a handful of target genes have been analyzed in the osteoclast lineage [6], [8]–[15]. Hundreds of miRNAs have been identified, and each miRNA can potentially regulate hundreds of mRNAs. To date, miRNA expression profiling in osteoclasts has been limited to a study examining bone marrow macrophages treated with RANKL for 24 hours or a study examining the immortalized cell line RAW264.7 treated with RANKL for 1 or 3 days [8], [16]. Osteoclastogenesis is a complex, multistage process with distinct cellular phenotypes evident in the early, middle and late stages of differentiation. To understand how miRNAs control osteoclast formation and function, a critical first step is the identification of miRNAs that are expressed in these distinct stages of the differentiation, using a physiologically relevant culture system.

In the present study, we profiled miRNA expression during the early, middle, and late stages of osteoclastogenesis, in a population of primary murine bone marrow cells enriched for osteoclast progenitors. Clusters of differentially expressed miRNAs were identified, and computational target prediction tools and pathway analysis were used to identify sets of miRNAs that could regulate pathways critical for cell motility, cell-matrix interactions, and regulation of the actin cytoskeleton. This data set is a unique resource for future studies aimed at understanding the function of each miRNA that is regulated during osteoclast differentiation.

## Materials and Methods

### Ethics statement

All animal protocols were approved by the Institutional Animal Care and Use Committee at the University of Connecticut Health Center (protocol 100435-0315).

### Cell culture

Primary osteoclast precursor cultures were established using bone marrow from 6–8 week old C57BL/6 male mice, which had been enriched for osteoclast precursors by depletion of B220/CD45R-positive and CD3-positive cells (B and T lymphocytes, respectively). Briefly, bone marrow was isolated from femurs, tibias, and humeri, and depleted of erythrocytes by treatment with ammonium-chloride-potassium (ACK) buffer (Gibco Life Technologies, Grand Island, NY) [17]. Cells were incubated with Phycoerythrin (PE)-conjugated primary antibodies for CD45R and CD3 (eBioscience, San Diego, CA), and with magnetically labeled anti-PE microbeads (MiltenyiBiotec, Auburn, CA). Magnetic-Activated Cell Sorting (MACS) Column Technology (MiltenyiBiotec, Auburn, CA) was used to capture CD45R and CD3 positive cells in the column, and the flow-through contained a population of cells enriched for monocytic and non-lymphoid lineage cells. Flow cytometric analysis was performed to analyze the presence of CD45R and CD3 positive cells. Standard staining procedures were used to label the cells for flow cytometry. Nonviable cells were identified by their ability to incorporate propidium iodide (PI). Flow cytometry was performed using a BD-FACS Aria (BD Biosciences, San Jose, CA, USA), and data were analyzed using FlowJo software from Tree Star Inc (Ashland, OR, USA).

Cells were cultured in α-MEM (Gibco Life Technologies, Grand Island, NY) supplemented with 10% FBS (Fetal Bovine Serum, Atlas Biologicals, Fort Collins, CO) and 30 ng/ml murine recombinant M-CSF (eBioscience, San Diego, CA). Bone marrow-derived osteoclast precursor cells were cultured in the presence of 30 ng/ml M-CSF and 30 ng/ml murine recombinant RANKL (eBioscience) for up to 5 days [11].

### miRNA Microarray

Primary osteoclast precursors were plated at 500,000 cells/well in 6 well plates. Total RNA was isolated from differentiating cultures using the miRNeasy Mini kit (Qiagen, Valencia, CA). On-column DNase treatment was performed to reduce contamination with genomic DNA, and an additional treatment with RQ1 DNase (Promega, Madison, WI) was performed prior to gene expression analysis. RNA concentration and purity were assessed by spectrophotometric analysis. The quality of small RNAs in each sample was determined using the 2100 Bioanalyzer assay (Agilent Technologies, Santa Clara, CA).

Four independent cultures were analyzed for each time point. For each sample, 200 ng of total RNA were labeled using miRNA Microarray System with miRNA Complete Labeling and Hyb Kit (Agilent Technologies). According to the manufacturer’s instructions, the samples were hybridized for 20 hours onto a mouse miRNA Microarray, Release 15.0, 8×15 K (based on Sanger miRBase release 15.0), containing 627 mouse mature miRNA probes (Agilent Technologies). Hybridized and washed array slides were scanned at 5 µm resolution using an Agilent SureScan Microarray Scanner (Agilent Technologies). Image processing was completed using Feature Extraction Software (Agilent Technologies).

### Microarray data analysis

Microarray data were normalized and analyzed using the GeneSpring GX software (Technology 29152_v.17_0, Agilent Technologies). The complete data set may be accessed at NCBI-Gene Expression Omnibus (accession GSE53017). miRNAs detected in at least one sample were subjected to quantile normalization to allow comparison between the microarray chips, and relative expression is presented as log(base 2). One-way Analysis of Variance (ANOVA) was performed on miRNAs expressed during the time course of osteoclast differentiation analyzed (days 1, 3, and 5). miRNAs showing >2 or <−2 fold-change, with p<0.01, were considered statistically significant. A hierarchical clustering analysis was used to organize the genes based on similarities in their expression profiles. A list of putative miRNA targets was identified using the prediction algorithm DNA Intelligent Analysis (DIANA) DIANA-microT-CDS (v5.0). The predicted miRNA targets were annotated into functional pathways using DIANA-miRPath (v2.0) (http://diana.imis.athena-innovation.gr/DianaTools/index.php?r=site/index) [18].

### Quantitative Real time PCR

miRNA expression levels were analyzed using the TaqMan MicroRNA Assay (Life Technologies, Grand Island, NY). According to the manufacturer’s instructions, 22.5 ng of RNA were reverse transcribed with specific primers to generate cDNA. The expression of miR-365, miR-99b, and miR-451 was detected by qPCR in a MiQ qPCR cycler (Bio-Rad) and normalized to U6 small nuclear RNA (RNU6b) levels, using the absolute quantification method. Data are presented as mean ± SEM; N = 4. Results shown are representative of 2–3 independent experiments. Data were analyzed by one-way ANOVA with Bonferroni post-hoc test as appropriate (KaleidaGraph, Synergy Software, Reading, PA).

### miRNA inhibitor or mimic transfection and osteoclastogenesis

Bone marrow cells isolated from long bones of 6–8-week-old C57BL/6 mice were cultured overnight, to reduce the mesenchymal cell population. Enrichment in monocyte precursor population was achieved by subjecting the non-adherent cells to density gradient centrifugation with Ficoll (GE Healthcare, Piscataway, NJ). Bone marrow-derived monocytes (BMMs) were plated at 30,000 cells/well in 96-well plates and cultured in α-MEM media supplemented with 10% FBS in presence of 10 ng/ml M-CSF for 2 days. Cells were then transfected with 50 nM miRNA inhibitors or mimics (Dharmacon), or the appropriate scrambled control, using HiPerFect transfection reagent (Qiagen, Valencia, CA). Six hours post-transfection, osteoclast differentiation was induced with M-CSF and RANKL treatment (30 ng/ml each) [11].

### In Vitro Osteoclast Formation Assay

Cells were fixed in 2.5% glutaraldehyde in PBS, and TRAP activity was detected according to the manufacturer’s instructions using the Acid Phosphatase Leukocyte (TRAP) kit (Sigma-Aldrich). Osteoclast cultures were imaged using light microscopy and TRAP positive cells with more than 3 nuclei were counted as osteoclasts. CellSens Dimension software (Olympus) was used to measure osteoclast area; N = 6. Data shown are representative of 2 independent experiments.

## Results and Discussion

### Differential miRNA expression during in vitro osteoclastogenesis

**Figure 1:**
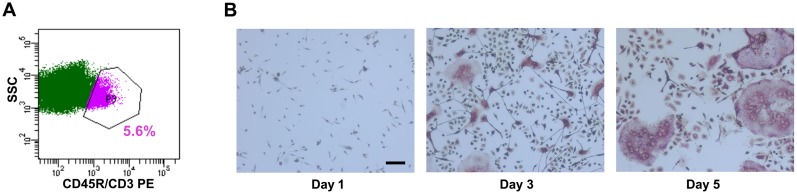
MACS sorting depleted lymphocytes from bone marrow-derived osteoclast cultures. (A) Bone marrow cells were depleted of CD45R^+^ and CD3^+^ cells by MACS sorting, using CD45R and CD3 antibodies. The negative population, constituted by non-lymphoid cells and enriched for monocytes, was collected and analyzed by flow cytometry. The gated population identifies the CD45R/CD3^+^ cells (purple) (n = 3). (B) The enriched population of bone marrow-derived osteoclast precursors was cultured in the presence of M-CSF and RANKL (30 ng/ml each) for up to 5 days. Representative images of TRAP stained cultures after 1, 3, and 5 days of differentiation were captured using 10X magnification. (n = 3).

Mouse bone marrow is a widely used source of primary osteoclast progenitors for in vitro analyses. However, it represents a highly heterogeneous population, containing monocytes, megakaryocyte precursors, macrophages, neutrophils, and a higher percentage of lymphocytes, which can interfere with the study of osteoclast-specific mechanisms. Our unpublished data indicate that, after erythrocyte depletion, 25–30% of total bone marrow is B220 (CD45R)^+^ and 5–7% is CD3^+^ (unpublished data). In addition, the majority of the most efficient osteoclast precursors is contained within the B220^−^/CD3^−^/CD11b^−/lo^ population in the bone marrow [19], [20]. Therefore, we sought to decrease the heterogeneity of the precursor population, prior to the initiation of osteoclastogenesis, by depleting the lymphocytic cells (B220^+^ or CD3^+^). Mouse bone marrow cells were subjected to MACS sorting using CD45R and CD3 antibodies. Flow cytometric analysis confirmed a 93–95% depletion of T and B cells, thereby decreasing the heterogeneity of the marrow cells that were subsequently plated for experiments (Figure 1A). We cultured mouse bone marrow-derived osteoclast precursors in the presence of M-CSF and RANKL for up to 5 days. At days 1, 3, and 5 of culture, osteoclast formation was monitored by TRAP staining, and total RNA was harvested (Figure 1B). Under these conditions, osteoclast number increased progressively, and mRNA levels for the osteoclast markers TRAP and Cathepsin K were increased from day 1 to day 3, and maintained at day 5, as we previously reported [11].

The Agilent mouse miRNA microarray that we used contained 627 probes for mature miRNAs. In our sample set, 258 miRNAs were significantly detected in at least one time point. 142 of these miRNAs were significantly changed during the time course investigated, and several were identified for the first time in the osteoclast lineage. Among the significantly changed miRNAs, 93 miRNAs showed >±2 fold-change. 49 miRNAs were up-regulated over time, whereas 44 were down-regulated (Figure S1). Based on their level of expression and change during differentiation, hierarchical clustering divided these 93 miRNAs into 7 clusters (Table 1, Figures 3–9, Figure S1).

**Table 1:** miRNA clusters.

Cluster number	Name/Description
1	Highly expressed
2	Modestly expressed down-regulated
3	Modestly expressed up-regulated
4	Well expressed up-regulated
5	Well expressed down-regulated
6	Most down-regulated over time
7	Most up-regulated over time

Hierarchical clustering of the miRNAs significantly changed during osteoclast differentiation generated 7 subgroups.

### Verification of microarray results

We first compared our microarray results with other published murine miRNA profile analyses in osteoclastic cultures. The expression pattern that we observed for 17 of the significantly regulated miRNAs was similar to that observed in a 24 or 82 hour RANKL-driven RAW264.7 cell differentiation, or in a 24-hour RANKL-treatment of bone marrow macrophages (Table 2) [8], [16]. However, a few discrepancies were noted in miRNA expression trends between our microarray data and those published by other investigators. These are likely attributable to differences in the experimental design; most notably differences in the percentage of osteoclast precursor cells used, as well as the time in culture. Our study is unique in that we analyzed an enriched population of primary osteoclast precursors from the bone marrow, and evaluated miRNA expression during early, middle, and late phases of differentiation.

**Table 2:** Changes in miRNA profile during murine osteoclastogenesis, confirmed by other microarray studies.

miRNA	Expression during osteoclastogenesis	Experimental system	References
let-7a-5p	↑	BMMs, RAW264.7	[8], [16]
let-7e-5p	↑	BMMs, RAW264.7	[8], [16]
let-7f-5p	↑	BMMs, RAW264.7	[8], [16]
miR-100-5p	↑	RAW264.7	[16]
miR-125a-5p	↑	RAW264.7	[16]
miR-125b-5p	↑	RAW264.7	[16]
miR-146a-5p	↑	RAW264.7	[16]
miR-146b-5p	↓	RAW264.7	[16]
miR-185-5p	↑	RAW264.7	[16]
miR-29b-3p	↑	BMMs, RAW264.7, bonemarrow-derived osteoclastprecursors	[8], [11], [16]
miR-338-3p	↓	RAW264.7	[16]
miR-365-3p	↑	RAW264.7	[16]
miR-378-3p	↑	RAW264.7	[16]
miR-674-3p	↑	RAW264.7	[16]
miR-689	↓	RAW264.7	[16]
miR-98-5p	↑	BMMs, RAW264.7	[8], [16]
miR-99a-5p	↑	RAW264.7	[8], [16]

Published studies in RAW264.7 cell line and mouse bone marrow macrophages (BMMs) confirm the expression pattern for 17 of the 97 miRNAs regulated during the course of osteoclastogenesis [8], [16].

**Figure 2:**
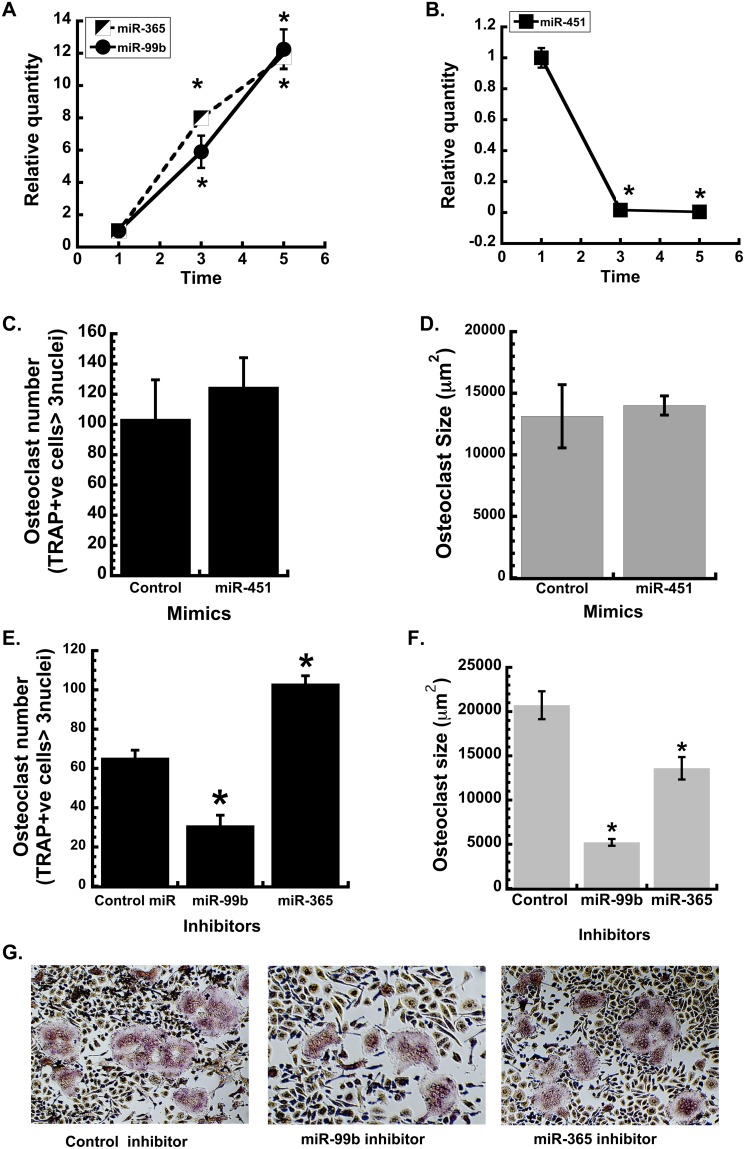
Validating the expression and function of candidate miRNAs from the microarray analysis. The expression of selected miRNAs, that were changed >±2 fold during osteoclast differentiation, was confirmed by qRT-PCR. (A) miR-365 and miR-99b levels, and (B) miR-451 levels were quantified after 1, 3, and 5 days of differentiation with M-CSF and RANKL (30 ng/ml each). Gene expression was normalized to U6. n = 4; * significantly different from day 1 (p<0.01). Primary BMMs were transfected with 50 nM miRNA mimic (C and D) or miRNA inhibitors (E and F) or the appropriate non-targeting control. Cells were differentiated into osteoclasts with treatment of M-CSF and RANKL (30 ng/ml each) for 3 days. Osteoclast formation was evaluated by TRAP staining. Osteoclast number and size, *p<0.05 different from control inhibitor (n = 6). (G) Representative images of miR-99b, −365 and control inhibitor transfected osteoclasts (4x magnification).

We next verified changes in the expression of several miRNAs using quantitative RT-PCR (qRT-PCR), focusing on 3 miRNAs that had not been previously identified, to our knowledge, in cells of the osteoclast lineage: miR-99b-5p, miR-365-3p and miR-451. In our data set, miR-365-3p was well expressed and highly up-regulated (Cluster 4), whereas miR-99b-5p was less well expressed, but one of the most prominently up-regulated miRNAs during osteoclast differentiation (Cluster 7). In contrast, miR-451 was well expressed in precursors, but strongly down-regulated during differentiation (Cluster 5). qRT-PCR verified the results of the array data (Figure 2). Expression of miR-99b-5p and miR-365-3p increased 12 fold between days 1 and 5 of culture (Figure 2A). Expression of miR-451 decreased dramatically between 1 and 3 days of culture, such that it was virtually undetectable on days 3 or 5.

Since miR-451 levels were dramatically decreased during osteoclast differentiation, to test its function, BMMs were transfected with a miR-451 mimic prior to RANKL-mediated differentiation. However, osteoclast number and size were not affected by the miR-451 mimic, suggesting that this miRNA may not have a significant role in osteoclastogenesis in vitro, although these data do not preclude potential actions in vivo (Figure 2C and D). It has been reported that miR-451 is required for erythroid differentiation and homeostasis [21], [22]. Therefore, it is possible that the high levels of miR-451 detected at day 1 are due to the presence of erythrocyte precursors in the cultures, which will not survive in culture with M-CSF and RANKL. Indeed, we could not detect expression of miR-451 by qRT-PCR in the bipotential mouse monocytic cell line RAW264.7 (data not shown).

To determine whether the strongly up-regulated miRNAs had a positive role in osteoclastogenesis, bone marrow-derived monocytes (BMMs) were transiently transfected with inhibitors for miR-99b-5p or miR-365-3p. Osteoclast number and size were determined after 3 days of culture in the presence of RANKL. Inhibition of miR-99b activity resulted in a 50% decrease in osteoclast number compared to the control inhibitor, and the osteoclast size was also significantly reduced (Figure 2E–G). This decline in osteoclast number and size indicates that miR-99b is crucial for osteoclast formation.

Previously, miR-99b was shown to be up-regulated in dendritic cells and monocytes during inflammation [23], [24]. In other cell types, miR-99b was shown to directly target components of the TNFα (tumor necrosis factor α) signaling pathway: *Tnfα*, *Tnfrsf4* (Tumor Necrosis Factor Receptor Superfamily, Member 4), and *Traf2* (TNF receptor-associated factor 2) [23], [25]–[27]. In addition, the miR-99 family has been shown to directly target *Mtor* (mammalian target of rapamycin) [28], [29]. Since TNFα signaling and the mTOR pathway are both key positive regulators of osteoclastogenesis, it is possible that miR-99b could play a role in fine tuning and integrating the response to TNFα and mTOR signaling, to promote optimal osteoclast differentiation. It is also likely that other yet undiscovered miR-99b targets are important for osteoclastogenesis.

In contrast to miR-99b, inhibition of miR-365 increased osteoclast number, while decreasing osteoclast size. These data suggest that miR-365 may fine tune osteoclastogenesis, regulating osteoclast size and number in an opposing manner. In other cell types, miR-365 has been shown to target Cyclin D1 and CDC25A, as well as pro-apoptotic BAX [30], [31]. Inhibition of miR-365 activity could lead to increased cell number, a potential explanation for the increased osteoclast number observed in our studies (Figure 2). The increased levels of miR-365 during osteoclast differentiation could slow proliferation and increase survival.

Notably, miR-99b is transcribed in an evolutionary conserved cluster that includes let-7e and miR-125a, all of which were significantly up-regulated during osteoclastogenesis, as assessed by our microarray (Figure 5A) [32]. Little is known about the mechanisms regulating the transcription of the miR-99b∼let-7e∼miR-125a cluster. In contrast, miR-365 is transcribed from two independent genetic loci (on mouse chromosomes 11 and 16), and the expression of miR-365 from the chromosome 16 locus is activated by Sp1 and NFκB, two transcription factors that promote osteoclastogenesis [33], [34]. It may be of interest to determine if only one miR-365 locus or both loci contribute to the miR-365 signal detected during osteoclast differentiation. Overall, identifying other mRNA targets of miR-365 and −99b will be critical for understanding the signaling cascades regulated by these miRNAs in osteoclasts.

### Target prediction and correlated pathways for miRNA expression clusters

miRNAs fine tune cellular responses, and osteoclastogenesis is a finely orchestrated process. A single miRNA has many targets, and a single mRNA can contain binding sites for several different miRNAs. One miRNA can regulate families of structural or signaling molecules within a particular pathway, amplifying or dampening the effects of extracellular signals, to balance and buffer cellular responses, as well as regulating the cross talk between signaling pathways [35]–[37]. Because of this, integrating pathway analysis of potential miRNA targets with miRNA expression data is one way to distill a complex data set, and suggest specific pathways that could be enriched in targets of regulated miRNAs.

**Figure 3:**
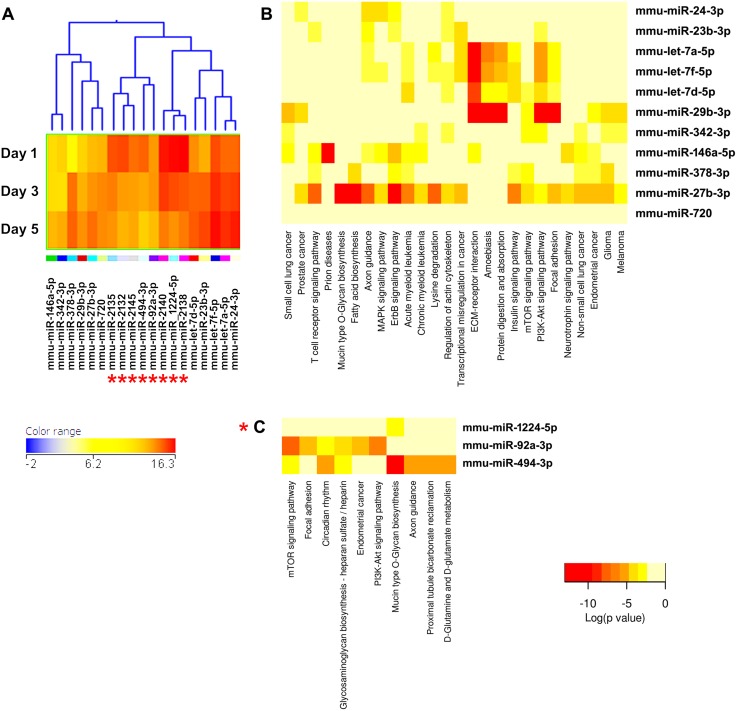
Cluster 1, highly expressed miRNAs during osteoclastogenesis. (A) miRNA expression heat map. The dendrogram shows the similarities between the expression profiles of the significantly changed miRNAs. Down-regulated miRNAs are indicated by the “*” symbol. Blue represents low expression, red high expression, and yellow intermediate expression. Please note that, for this cluster only, the color balance of the expression heat map and its key have been altered (compared to the original Cluster 1 in Figure S1), to better illustrate the changes in expression with time of differentiation. (B) Up-regulated miRNAs and predicted pathways heat map. Red color indicates lower p values (more significant), and higher interaction of each miRNA with a specific molecular pathway. (C) Down-regulated miRNAs and predicted pathway heat map. Significant miRNA-pathway interaction p<0.001.

**Figure 4:**
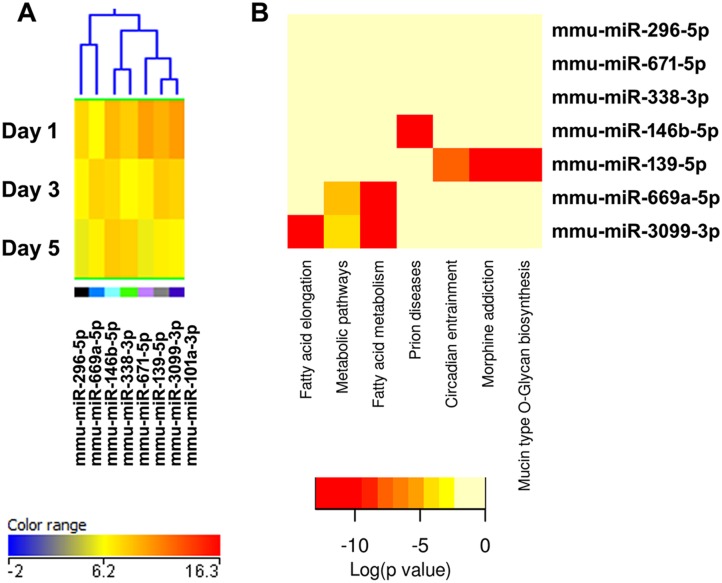
Cluster 2, modestly expressed miRNAs down-regulated during osteoclastogenesis. (A) miRNA expression heat map. Blue represents low expression, red high expression, and yellow intermediate expression. (B) Predicted pathways heat map. Red color indicates lower p values.

**Figure 5:**
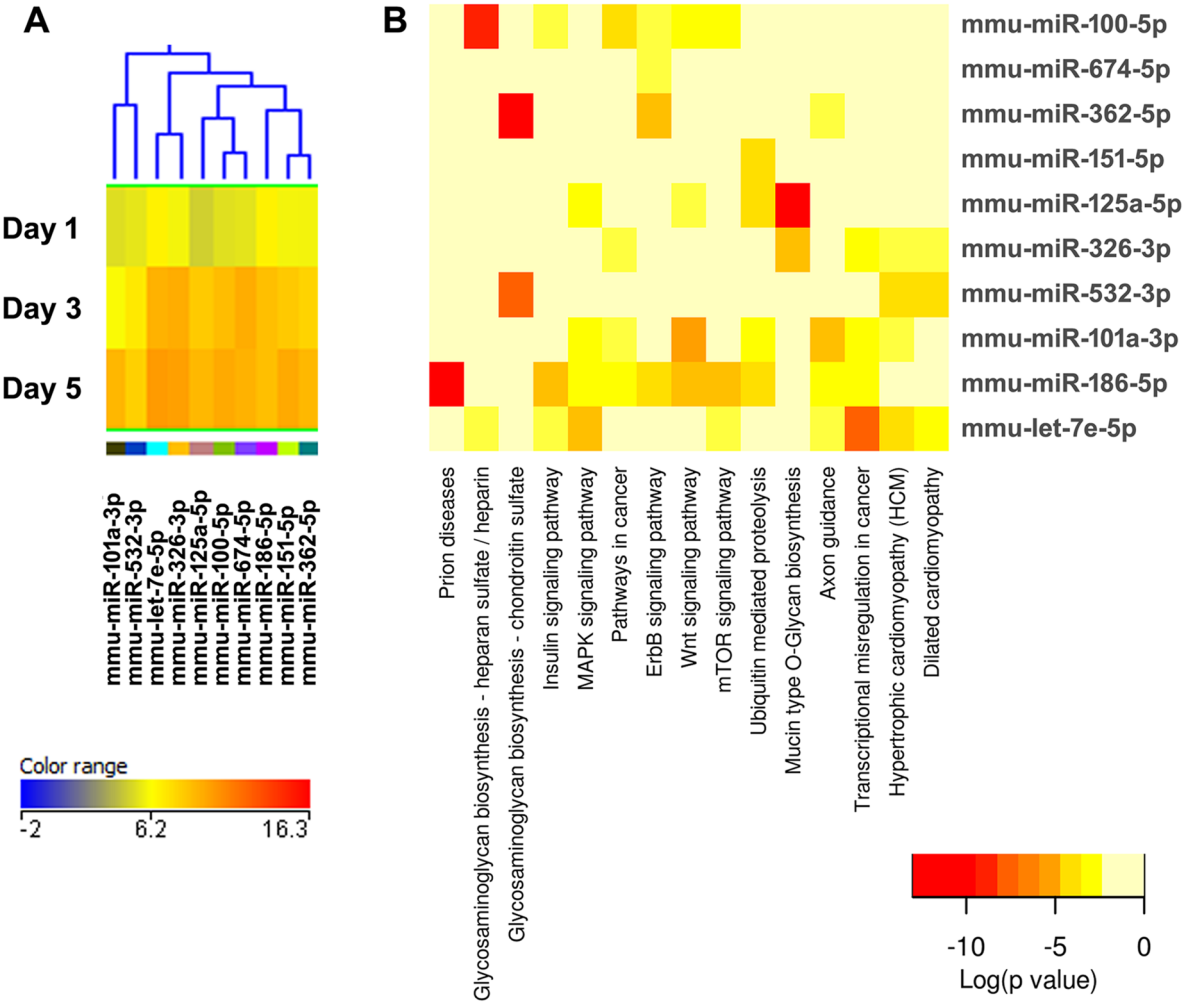
Cluster 3, modestly expressed miRNAs up-regulated during osteoclastogenesis. (A) miRNA expression heat map. Blue represents low expression, red high expression, and yellow intermediate expression. (B) Predicted pathways heat map. Red color indicates lower p values.

**Figure 6:**
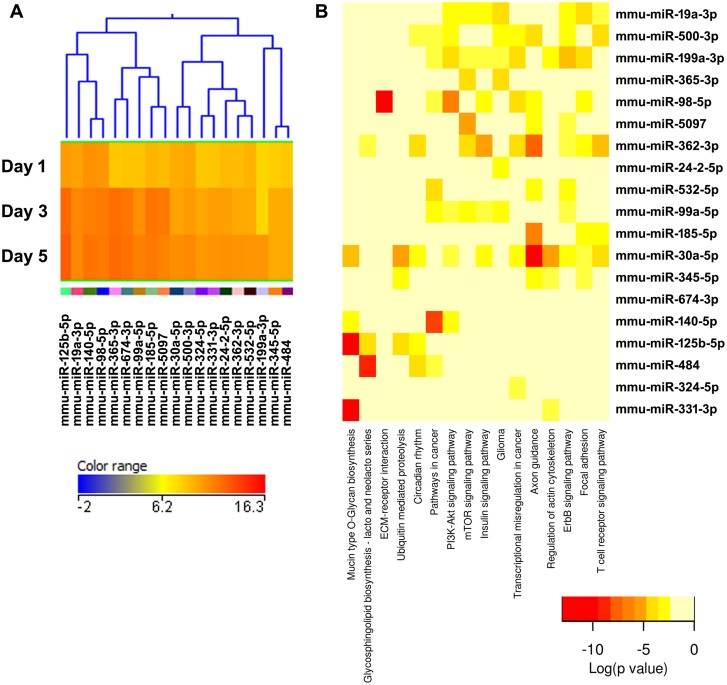
Cluster 4, well expressed miRNAs up-regulated during osteoclastogenesis. (A) miRNA expression heat map. Blue represents low expression, red high expression, and yellow intermediate expression. (B) Predicted pathways heat map. Red color indicates lower p values.

**Figure 7:**
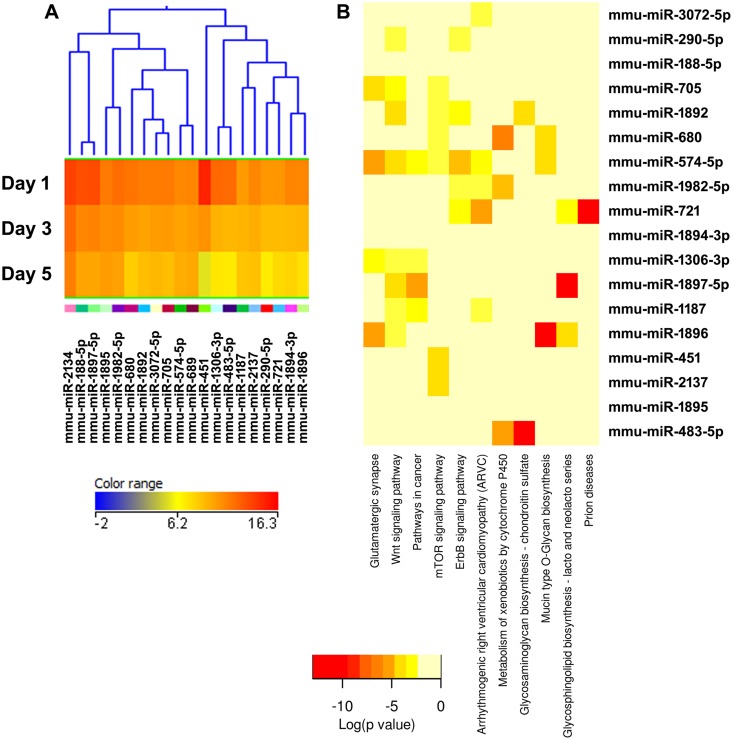
Cluster 5, well expressed miRNAs down-regulated during osteoclastogenesis. (A) miRNA heat map. Blue represents low expression, red high expression, and yellow intermediate expression. (B) Predicted pathways heat map. Red color indicates lower p values.

**Figure 8:**
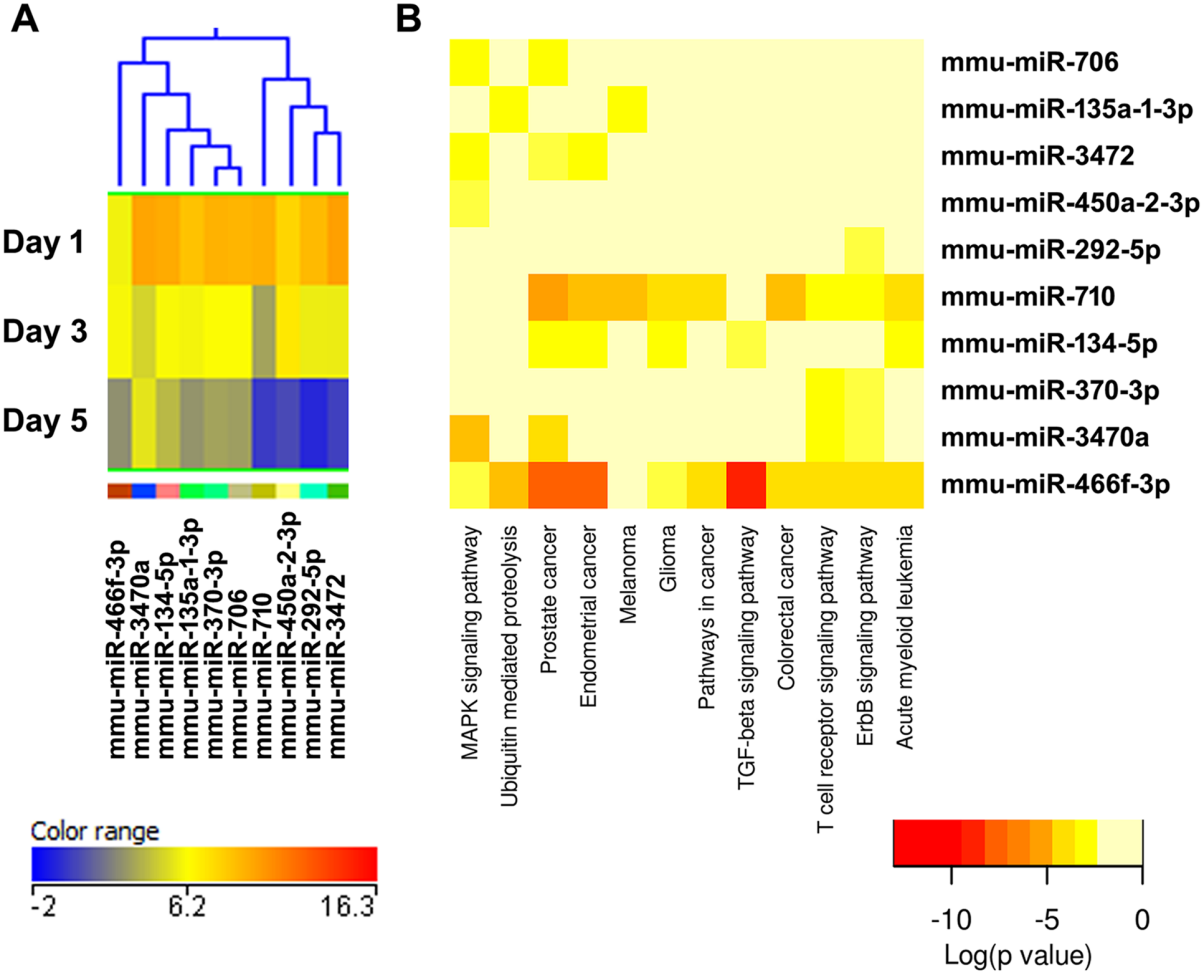
Cluster 6, miRNAs most down-regulated during osteoclastogenesis. (A) miRNA expression heat map. Blue represents low expression, red high expression, and yellow intermediate expression. (B) Predicted pathways heat map. Red color indicates lower p values.

**Figure 9:**
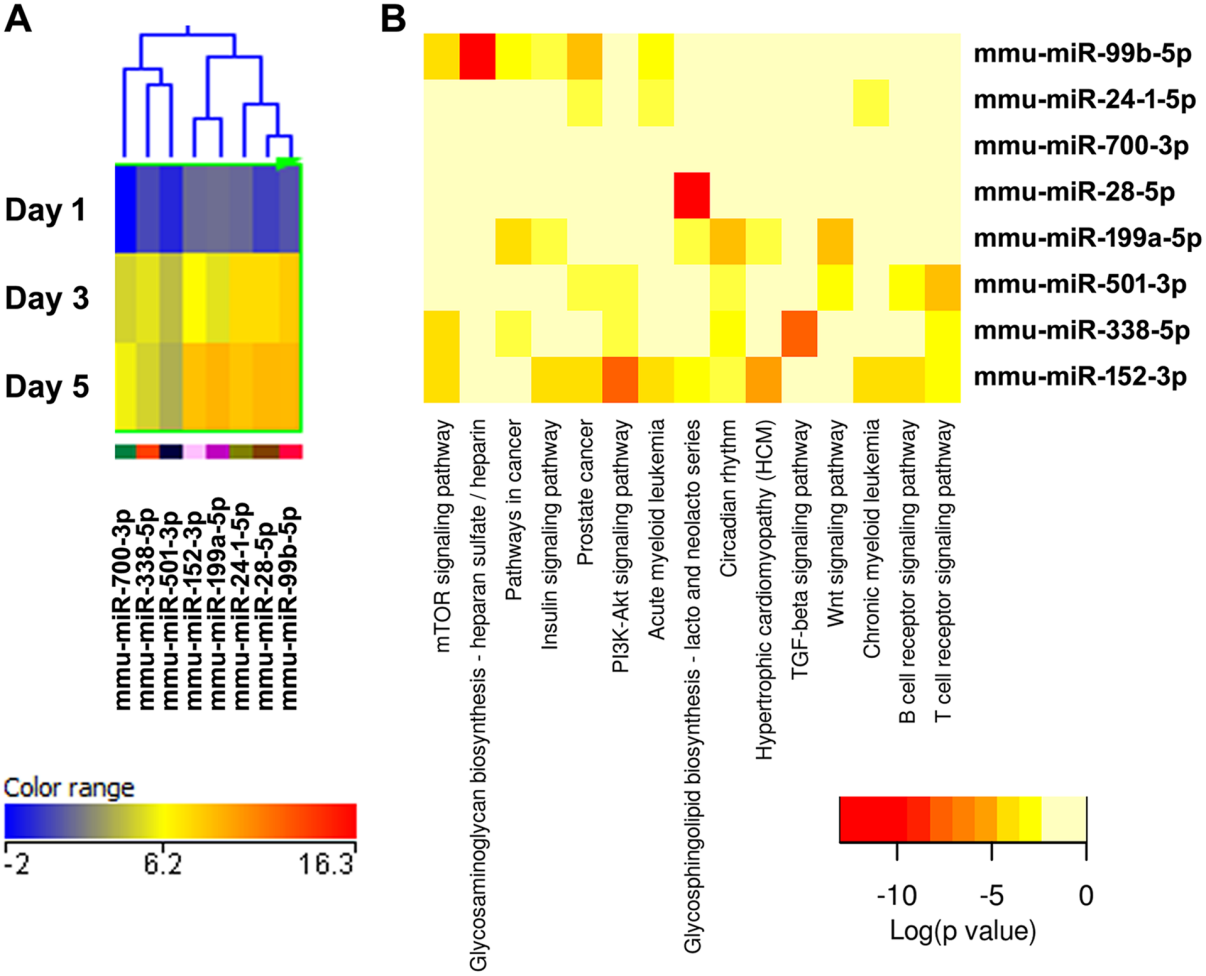
Cluster 7, miRNAs most up-regulated during osteoclastogenesis. (A) miRNA expression heat map. Blue represents low expression, red high expression, and yellow intermediate expression. (B) Predicted pathways heat map. Red color indicates lower p values.

Therefore, we performed a computational target prediction analysis coupled to pathway analysis using DIANA-miRPath (v2.0) software [18]. Potential target RNAs for up- or down-regulated miRNAs from each cluster were identified using the prediction algorithm DIANA-microT-CDS (v5.0). This algorithm recognizes potential miRNA binding sites located in the coding sequence and in the 3′ UTR of an mRNA, based on complementary pairing with nucleotides in position 1–9 at the 5′ end of the miRNA (i.e. the seed binding region). Additional features taken into consideration include conservation of the sequence element across species and accessibility of the target site [38], [39]. The potential miRNA targets for each miRNA cluster (Figures 3–9) or for the entire set of up or down-regulated miRNAs (Figures S2 and S3) were then subjected to KEGG (Kyoto Encyclopedia of Genes and Genomes) pathway analysis. The DIANA-miRPath software calculates the significance for all the miRNA-mRNA pairs in a pathway, and then combines them into a merged P-value for each pathway [18]. The results are reported as heat maps, and the pathways are clustered based on significance levels. The more intense red color indicates higher probability that a specific pathway is significantly enriched with target genes for a certain miRNA.

Cluster 1 is composed of highly expressed miRNAs, and contains miRNAs that are both up- and down- regulated during osteoclastogenesis. Therefore, separate pathway analysis was performed for the up- and down- regulated miRNAs. Among the highly expressed and up-regulated miRNAs is miR-29b, a member of the miR-29 family (Figure 3A). We and others previously showed that expression of all miR-29 family members increases during osteoclastogenesis [11], [16]. In addition, we demonstrated that miR-29 promotes osteoclast commitment and migration, and is critical for osteoclast formation. We validated several novel miR-29 targets in the osteoclast lineage, including calcitonin receptor. Further, miR-29 targets genes important for the macrophage lineage, *Nfia* (Nuclear factor 1/A), *Cd93*, and *Gpr85* (G protein coupled receptor 85); as well as genes modulating cell migration, including *Cdc42* (Cell division control protein 42) and *Srgap2* (SLIT-ROBO Rho GTPase activating protein 2) [11]. With regard to miR-29b, the pathway analysis suggests that functions such as cell-matrix interactions, focal adhesion, and PI3 K/Akt to be most significantly associated with miR-29 (Figure 3B). Several predicted miR-29 targets within these pathways have been experimentally confirmed. These include mRNAs for a large number of extracellular matrix proteins (collagens, laminins), the tumor suppressor *Pten*, *Igf1* (insulin growth factor 1), and *Mcl1* (myeloid cell leukemia 1) [40]–[43]. Overall, these observations suggest that the pathway analysis for targets of individual miRNAs is, at least in part, validated by experimental data.

The validity of this pathway clustering approach is further supported by analysis of the miRNA family formed by miR-99a, miR-99b, and miR-100. Each these miRNAs is up-regulated during the course of osteoclastogenesis, although with different amplitude. miR-99a-5p belongs to expression cluster 4, miR-99b-5p to cluster 7, and miR-100-5p to cluster 3 (Figures 5A, 6A, and 9A). KEGG pathway analysis predicted, with a high degree of confidence (p<0.001), miR-99b regulation of the mTOR (mammalian target of rapamycin) pathway, whereas association of miR-99a and miR-100 with the mTOR pathway was predicted with lower confidence (p<0.005) (Figures 5B, 6B, and 9B). As discussed above, several studies demonstrated the role of the miR-99 family in repressing mTOR signaling in different cell systems, including wound healing keratinocytes, as well as prostate, endometrial, and pancreatic cancer cells [28], [29], [44], [45]. In our study, numerous clusters of miRNAs, both up- and down-regulated during the course of osteoclast differentiation, were predicted to target components of the mTOR pathway. Cluster 4 appears to be particularly enriched in miRNAs with potential targets in the mTOR pathway (Figure 6B).

In osteoclasts, mTOR has been implicated in the regulation of apoptosis. Crucial signaling pathways triggered by M-CSF, RANKL, and TNFα converge on the activation of S6 K (p70 ribosomal protein S6 kinase), a main effector of the mTOR signaling cascade. By regulating the process of translation, mTOR promotes osteoclast differentiation, survival, and bone-resorbing activity [46], [47]. Although miRNA-mediated modulation of mTOR factors has been widely investigated in other biological systems, this represents a novel area of research in the bone field.

KEGG pathway analysis showed that several miRNAs up-regulated during osteoclast differentiation (clusters 1, 3, and 4) were predicted to target extracellular matrix-receptor interactions, regulators of the actin cytoskeleton, focal adhesion, and axon guidance (Figures 3B, C, 5B, 6B, Figure S2). Osteoclasts do not use focal adhesions to adhere to the bone surface. However, several proteins belonging to the KEGG functional pathway for focal adhesion participate in the formation of podosomes and actin rings, which are critical for osteoclast adhesion. These include integrins and proteins of the Rho GTPase signaling pathway. Similarly, although axon guidance is usually studied in regard to neuronal development, numerous factors within this KEGG pathway play an essential role in the osteoclast lineage, such as ephrins, semaphorins, and Rho GTPases [48]–[50]. Moreover, many signaling cascades that regulate focal adhesion and axon guidance, as well as extracellular matrix-receptor interaction, converge on reorganization of the actin cytoskeleton. This is a key process that regulates a variety of biological functions, including cell motility, morphology, and attachment, as well as gene expression, differentiation, and apoptosis. Cytoskeletal remodeling and cell migration are fundamental for osteoclast formation and bone resorption activity, and they are tightly controlled at multiple levels [51]. We and others have previously shown that miRNAs can modulate osteoclast motility and activity [9]–[11]. However, a complete understanding of the miRNAs involved in fine tuning the regulation of cytoskeletal reorganization is lacking. Our study contributes to the identification of miRNAs that may play a role in this function.

## Conclusions

Documentation of how individual miRNAs are regulated during the course of osteoclastogenesis is a critical step in understanding the role of miRNAs in this complex process. Expression arrays are excellent for this purpose, since they provide an indication of whether a particular miRNA is highly expressed or more modestly expressed, in relation to another. We detected 93 miRNAs significantly regulated during osteoclast differentiation. Although this seems like a small number of regulated entities, compared to the thousands of regulated genes often demonstrated by mRNA expression arrays, comprehending the function of the regulated miRNAs is complicated by the fact that each one has the potential to target tens or hundreds of mRNAs. Different miRNA target prediction algorithms have varying levels of false-positive and false-negative results. Since miRNAs often regulate gene families or several components of a particular signaling pathway, we believe that pathway analysis of potential targets could enhance the likelihood of validating relevant miRNA-target interactions. Moreover, the pathway analysis that we used can also provide information on whether an mRNA is predicted to be targeted by more than one regulated miRNA.

Osteoclastogenesis is an intricate multi-step process, initiating with the proliferation and commitment of mononucleated precursors, and culminating in the formation of large bone-resorbing polykaryons. In this study, we identified a profile of miRNAs differentially expressed in the early, middle and late stages of osteoclastogenesis in primary cultures and validated the function of 2 highly up-regulated miRNAs, miR-365 and −99b, during osteoclastogenesis. Pathways that may be enriched in targets regulated by miRNAs during osteoclastogenesis include interaction between cells and extracellular matrix, axon guidance, focal adhesion, and remodeling of the actin cytoskeleton. This study provides important information on miRNAs with the potential to regulate osteoclast differentiation. Validation of individual mRNA-miRNA interactions by biochemical methods or reporter assays remains vital to understanding the molecular mechanisms regulating osteoclastogenesis. This understanding is critical for the development of novel therapies for skeletal pathologies caused by alterations in bone resorption activity.

## Supporting Information

Figure S1 **miRNA expression profiles during osteoclastogenesis.** (A) Heat map of the 93 miRNAs showing >±2 fold-change over 5 days of osteoclast differentiation. Fold-change was calculated between day 1 and day 3, day 1 and day 5, and day 3 and day 5. Hierarchical cluster analysis on gene expression divided the miRNAs in 7 groups. Blue represents low expression, red high expression, and yellow intermediate expression. (B) Schematic overview of the microarray results.
(TIF)

Figure S2 **Predicted pathway analysis heat map for all miRNAs up regulated during osteoclastogenesis in** **vitro.** Red color indicates lower p values.
(TIF)

Figure S3 **Predicted pathway analysis heat map for all miRNAs down regulated during osteoclastogenesis in** **vitro.** Red color indicates lower p values.
(TIF)
