# Supplementary figures and images for: Pathway Analysis of MicroRNA Expression Profile during Murine Osteoclastogenesis
Source: PLoS One. 2014 Sep 15;9(9):e107262. doi: 10.1371/journal.pone.0107262 (PMC4164525; doi:10.1371/journal.pone.0107262)

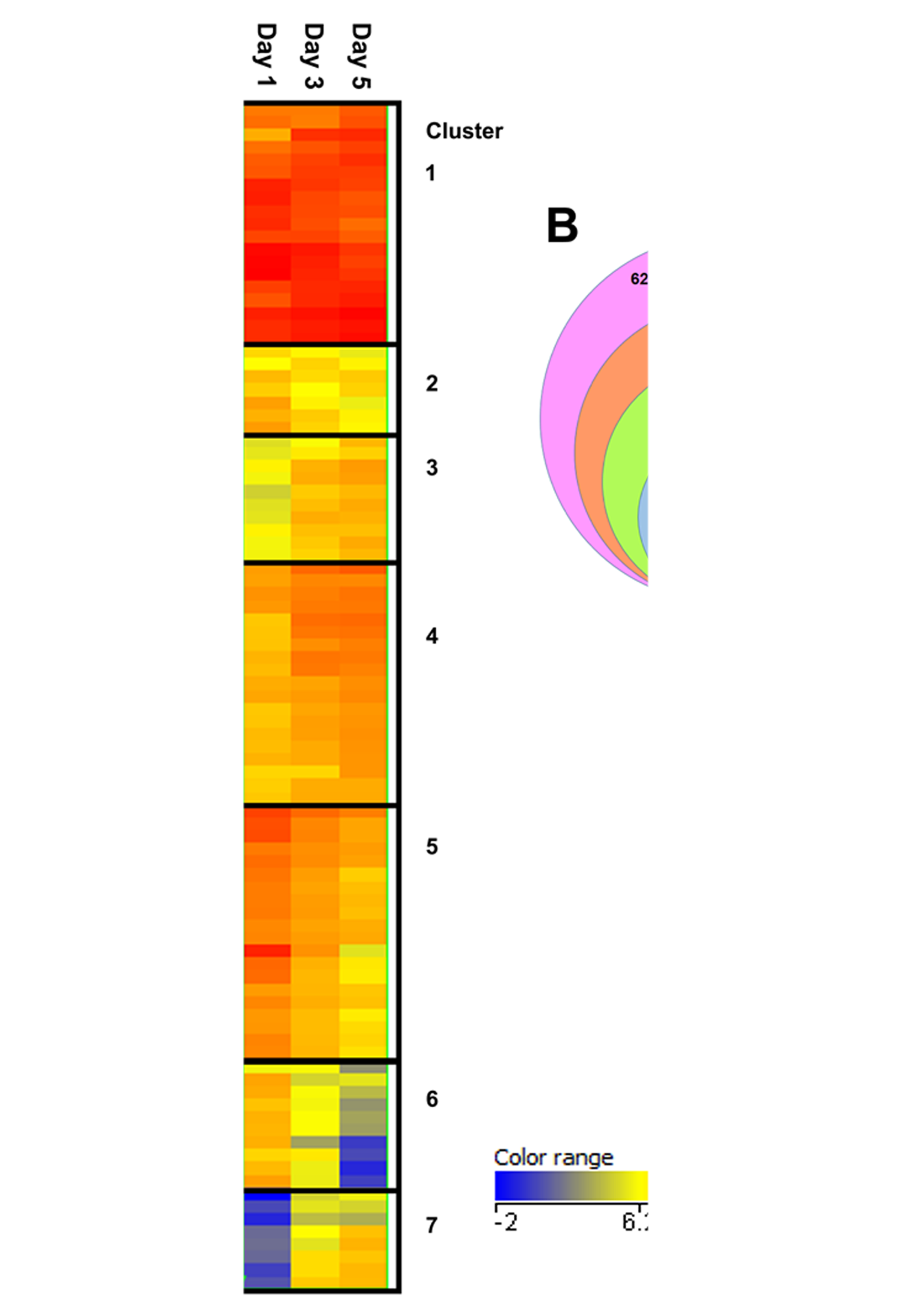

Supplement: Figure S1 — miRNA expression profiles during osteoclastogenesis. (A) Heat map of the 93 miRNAs showing >±2 fold-change over 5 days of osteoclast differentiation. Fold-change was calculated between day 1 and day 3, day 1 and day 5, and day 3 and day 5. Hierarchical cluster analysis on gene expression divided the miRNAs in 7 groups. Blue represents low expression, red high expression, and yellow intermediate expression. (B) Schematic overview of the microarray results. (TIF) [file pone.0107262.s001.tif]

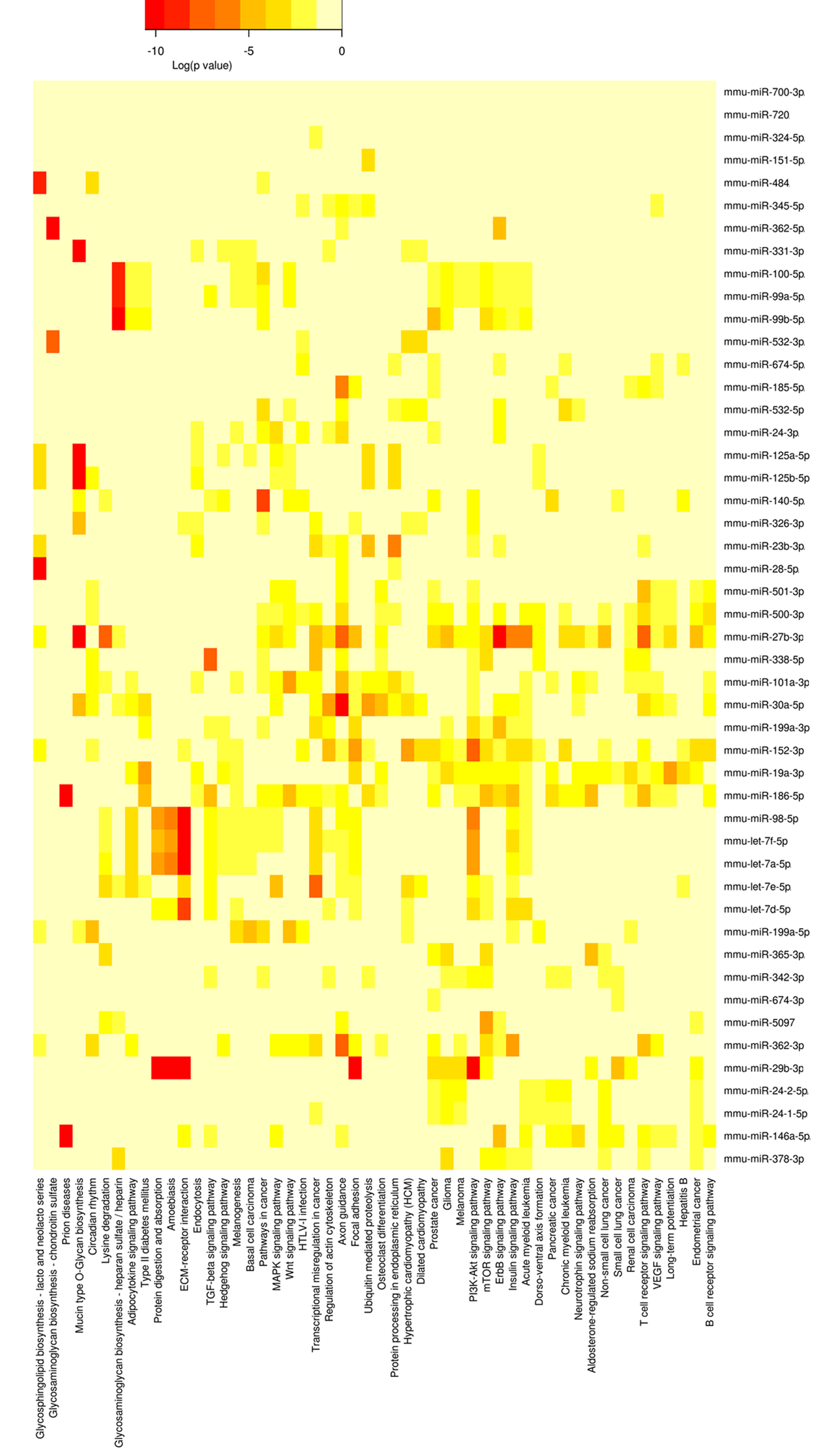

Supplement: Figure S2 — Predicted pathway analysis heat map for all miRNAs up regulated during osteoclastogenesis in vitro. Red color indicates lower p values. (TIF) [file pone.0107262.s002.tif]

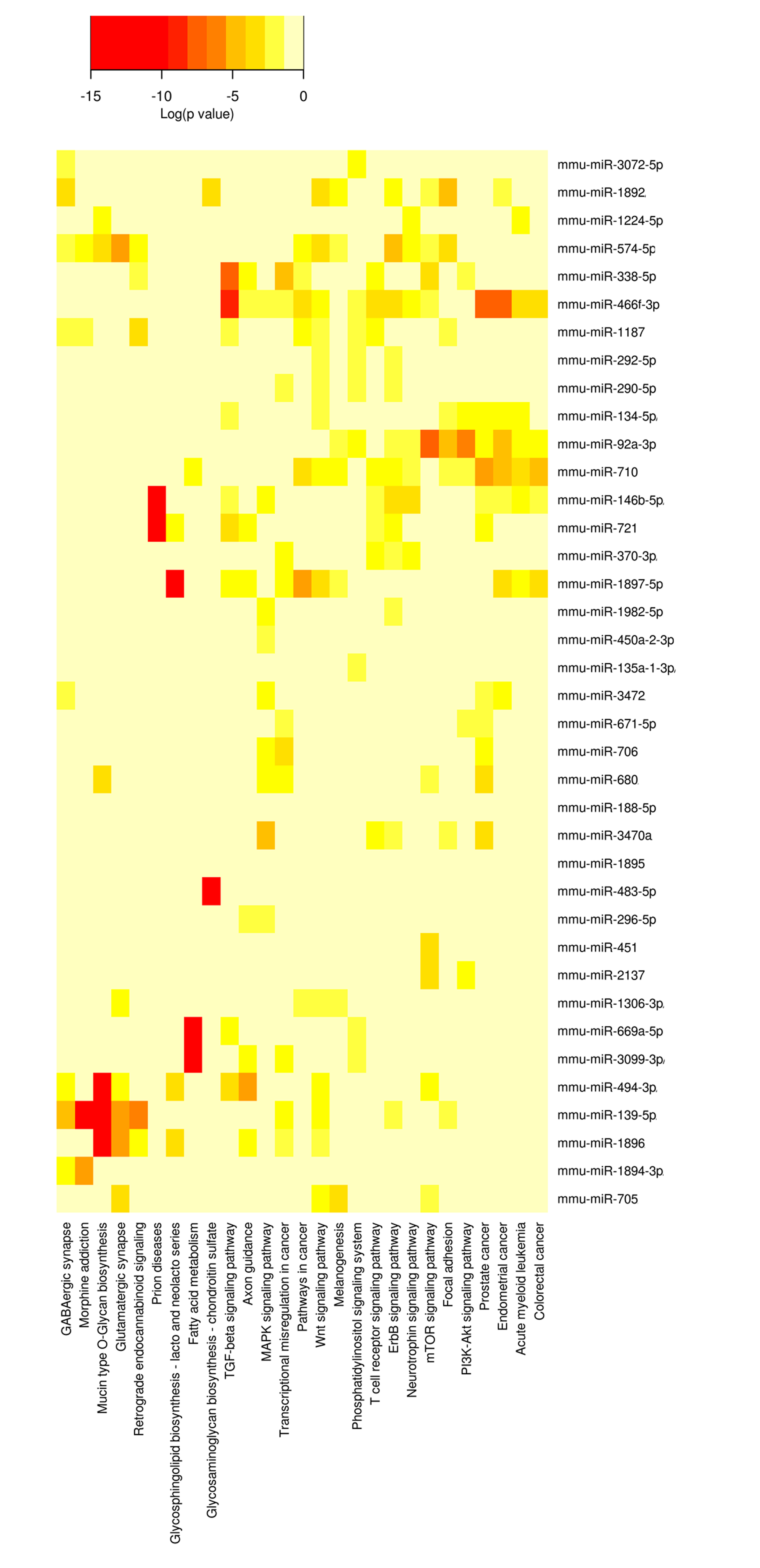

Supplement: Figure S3 — Predicted pathway analysis heat map for all miRNAs down regulated during osteoclastogenesis in vitro. Red color indicates lower p values. (TIF) [file pone.0107262.s003.tif]
